# Stat3 activation in human endometrial and cervical cancers

**DOI:** 10.1038/sj.bjc.6603597

**Published:** 2007-02-20

**Authors:** C-L Chen, F-C Hsieh, J C Lieblein, J Brown, C Chan, J A Wallace, G Cheng, B M Hall, J Lin

**Affiliations:** 1 Center for Childhood Cancer, Columbus Children's Research Institute, Columbus, OH, USA; 2 Ohio State Biochemistry Program, Columbus, OH, USA; 3 Graduate Program in Molecular, Cellular & Developmental Biology, Columbus, OH, USA; 4 Ohio State University Comprehensive Cancer Center, Ohio State University, Columbus, OH, USA

**Keywords:** Stat3, endometrial cancer, cervical cancer, tissue microarray

## Abstract

The activation of signal transducer and activator of transcription 3 (Stat3) has been implicated in the oncogenesis of cancer and is regarded as a novel target for cancer therapy. Stat3 is classified as a proto-oncogene, because an activated form of Stat3 can mediate oncogenic transformation in cultured cells and tumour formation in nude mice. The constitutive activation of Stat3 has been frequently detected in various types of human cancers. However, the constitutive activation of Stat3 in endometrial and cervical cancers has not been studied. We examined tyrosine phosphorylation of Stat3 (activated form of Stat3) in multiple endometrial and cervical cancer tissues using tissue microarray slides as well as cancer cell lines to explore the possible activation of Stat3. Our results indicated that elevated phosphorylation of Stat3 was detected in cervical and endometrial cancer cell lines. Our results also showed that elevated levels of phosphorylation of Stat3 protein were detected in the endometrial and cervical cancer specimens. This is the first study to demonstrate that Stat3 is activated in human endometrial and cervical cancer tissues. Immunohistochemical staining showed that activated Stat3 is associated with increased expression of downstream antiapoptotic genes, *Bcl-xL*, *survivin*, and *Mcl-1* in these tissues. Expression of a dominant-negative Stat3 mutant using adenovirus-mediated gene transfer inhibited cell growth and induced apoptosis in HeLa and SiHa cervical cancer cell lines expressing elevated levels of Stat3 phosphorylation. Further, a JAK/Stat3 small molecular inhibitor, JSI-124, induced apoptosis more selectively in HeLa and SiHa cancer cell lines than Ishikawa cell line without elevated levels of Stat3 phosphorylation. These results indicate that Stat3 is activated in human endometrial and cervical cancers and the inhibition of constitutive Stat3 signaling may be an effective target for cancer intervention in these two cancers.

Signal transducer and activator of transcription signaling pathways are activated in response to cytokines and growth factors (Darnell *et al*, 1994; Zhong *et al*, 1994). JAKs, Src, and epidermal growth factor receptor (EGFR) are some of the potential upstream activators of Stat3 (Sartor *et al*, 1997; Bromberg *et al*, 1998; Garcia *et al*, 2001). Stat3 is activated by phosphorylation at tyrosine (Tyr) residue 705, which leads to dimer formation, nuclear translocation, recognition of Stat3-specific DNA-binding elements, and activation of target gene transcription (Darnell *et al*, 1994; Zhong *et al*, 1994). A growing number of human malignancies and tumour formation are associated with high levels of activation of signal transducers and activators of transcription (STATs), very frequently Stat3 and Stat5 (Garcia *et al*, 1997; Garcia and Jove, 1998; Bowman *et al*, 2000). Stat3, as a major member of the STAT family consisting of Stat1, Stat2, Stat3, Stat4, Stat5*α*, Stat5*β*, and Stat6, plays important roles in cell differentiation and proliferation (Darnell *et al*, 1994; Schaefer *et al*, 1995; Buettner *et al*, 2002). Stat3 appears to be involved in the regulation of multiple systematic signal pathways and acts as a multi-functional protein. For example, Stat3 deletion in mice leads to embryonic lethality (Shen *et al*, 2004). It has been reported that Stat3 activation is sufficient to maintain an undifferentiated state of mouse embryonic stem cells (Niwa *et al*, 1998; Matsuda *et al*, 1999; Raz *et al*, 1999). In breast and hematopoietic cells, existing evidence demonstrate that Stat3 acts as an oncogenic protein and may be associated with chemotherapeutic resistance (Dalton and Jove, 1999; Rebbaa *et al*, 2001; Pollett *et al*, 2002; Real *et al*, 2002).

In a variety of human cancers, constitutive activation of Stat3 is sufficient to induce tumour formation (Bromberg *et al*, 1999; Buettner *et al*, 2002). The constitutive activation of Stat3 is frequently detected in a variety of human cancers (Bowman *et al*, 2000; Huang *et al*, 2000). *Stat3* has been classified as a proto-oncogene because an activated form of Stat3 can mediate oncogenic transformation in cultured cells and tumour formation in nude mice (Bromberg *et al*, 1999; Bowman *et al*, 2000). Constitutive Stat3 signaling may participate in oncogenesis by stimulating cell proliferation, promoting angiogenesis, mediating immune evasion, and conferring resistance to apoptosis induced by conventional therapies (Niu *et al*, 2002; Real *et al*, 2002; Wei *et al*, 2003; Wang *et al*, 2004). Inhibition of constitutively active Stat3 can induce apoptosis and inhibit cancer cell growth (Buettner *et al*, 2002), indicating constitutive Stat3 signaling is required for cancer cell survival and growth. Although the constitutive activation of Stat3 has been detected in various types of human cancers, there is no report on whether Stat3 is activated in endometrial cancer and cervical cancer tissues. Given that Stat3 can contribute to cancer progression and tumour angiogenesis, it is important to explore whether Stat3 is also activated in endometrial and cervical cancers. Therefore, we examined the Tyr phosphorylation of Stat3 at residue 705 (activated form of Stat3) in human endometrial and cervical cancer tissue microarray slides that contain multiple cancer specimens. Our results indicated that elevated levels of phosphorylation of Stat3 protein at Tyr residue 705 and serine residue 727 (Ser727) were detected in endometrial and cervical cancer specimens. These results suggest that activated Stat3 signaling may contribute to carcinogenesis in human endometrial and cervical carcinomas. We also showed that expression of a dominant-negative Stat3 mutant and a JAK/Stat3 small molecular inhibitor, JSI-124, induced apoptosis in HeLa and SiHa human cervical cancer cell lines expressing elevated levels of phosphorylated Stat3.

## MATERIALS AND METHODS

### Tissue microarray slides and immunohistochemistry

Human endometrial and cervical cancer tissue microarray slides were obtained from US Biomax Inc. (Ijamsville, MD, USA), Cybrdi Company (Frederick, MD, USA), and Imgenex Corporation (San Diego, CA, USA). These slides were baked at 60–65°C at various time periods according to the manufacturer's instruction, deparaffinised in xylene three times, transferred through two changes of 100% and 95% ethanol, and then rehydrated with graded ethanol. Antigen retrieval was done by boiling the slides in a small beaker filled with 10mM Sodium Citrate (pH 6.0) or 1mM EDTA (pH 8.0). Endogenous peroxidase activity was quenched by a 10-min incubation in 3% hydrogen peroxide. After antigen retrieval, the slides were washed two times in a 0.1% Tween/1 × TBS (0.1% TBST) bath (5 min each) at room temperature to remove nonspecific background binding. Protein blocking was performed by incubating the specimens in 5% normal goat serum or normal horse serum in 0.1% TBS for 1 h. The primary antibody was applied for 1 h at room temperature (1 : 30 dilution of anti-p-Stat3 (Y705) and (S727) antibody (Cell Signaling Technology, Beverly, MA, USA) and anti-Bcl-xL, -survivin (BD Sciences, Franklin Lakes, NJ, USA), and -Mcl-1 antibodies (Lab Vision, Corp. Fremont, CA, USA), in 0.1% TBST with normal serum or 1 : 50–100 dilution of antibodies that recognise Stat3 downstream targets in 0.1% TBST with normal serum. After a series of TBST rinses as described above, bound antibody was subsequently detected using a VETASTATIN ABC kit from VECTOR Laboratories, Inc. (Burlingame, CA, USA). For visualisations, the sections were then incubated with 3-amino-9-ethylcarbazole (AEC) high-sensitivity substrate chromogen from DakoCytomation (Carpinteria, CA, USA) for 2–30 min. Finally, the slides were counterstained with hematoxylin and mounted with CRYSTAL/MOUNT (Biomeda Corp., Foster City, CA, USA) for long-term preservation.

### Evaluation of immunohistochemical staining and statistical analysis

Immunostained slides were scored under microscope. The staining intensity was scored on the following scale: 0, no staining; 1, weak staining; 2, moderate staining; and 3, intense staining. Most or all of the cancer tissues showed staining in greater than 50% of the area. Scoring of the tissue microarray was completed by three independent researchers (JB, CC, and JL). Discrepant scores between three observers or researchers were re-scored to arrive at a single final score. As most of the normal tissues stained were scored as 0 but a few samples scored as 1, we therefore considered cancer samples with scores 2 and 3 were positive staining. The association of p-Stat3 (Tyr705) with the expression p-Stat3 (Ser727) and potential downstream genes was analysed using Pearson *χ*^*2*^ test.

### Cell lines and culture

Human endometrial cancer cell lines, RL95-2, Hec-1B and cervical cancer cell lines, C33A, HeLa, SiHa, and HT-3, and human papillomavirus E6/E7 (Ect1/E6E7)-immortalised cervical cell line were purchased from the American Type Culture Collection (ATCC). Ishikawa endometrial cancer cell line was kindly provided by Dr M Nishida. Hec-1B, C33A, HeLa, and SiHa were cultured in DMEM medium containing 10% fetal bovine serum (FBS), 100U/ml penicillin and 100 *μ*g/ml streptomycin (Invitrogen Life Technologies, Carlsbad, CA, USA). Ect1/E6E7-immortalised cervical cell line was cultured in keratinocyte-serum free medium containing 10% FBS, 100 U/ml penicillin and 100 *μ*g/ml streptomycin, 0.1 ng/ml EGF and 0.05 mg/ml bovine pituitary extract (Invitrogen Life Technologies, Carlsbad, CA, USA). RL95-2 cells were cultured in medium containing 10% FBS, 100 U/ml penicillin and 100 *μ*g/ml streptomycin, and supplemented with 0.005 mg/ml insulin (Invitrogen Life Technologies, Carlsbad, CA, USA). HT-3 cells were cultured in McCoy's 5a medium containing 10% FBS, 100 U/ml penicillin and 100 *μ*g/ml streptomycin (Biosource, Rockville, MD, USA). Cells were grown as attached monolayers and incubated in a humidified atmosphere with 5% CO_2_ at 37°C.

### Western blot

Cells were collected at 4°C in cold harvest buffer, supplemented with proteinase inhibitor cocktails and spun down at 3000 **g** for 5 min. Cell pellets were lysed in RIPA lysis buffer as described previously (Hsieh *et al*, 2005). Protein concentrations were quantitated using BCA protein assay kit from Pierce, Inc. (Rockford, IL, USA) according to the manufacture's protocol. Fifty or 100 *μ*g of cellular proteins were resolved on 10% PAGE gels in electrophoresis buffer and transferred to Hybond™-p membrane (Amersham Biosciences, Piscataway, NJ, USA) using transfer buffer with constant 100 V. The membranes were then blocked using 5% nonfat dry milk in TBST (Tris-HCl, pH 7.5, Tween, 0.1%) for 30 min at room temperature (RT) and were incubated with primary antibody over night at 4°C or for 1 h at RT using concentrations recommended by the manufacturer. The membranes were washed three times in 1 × TBST for 5 min each time. Proteins of interest were visualised using ECF™ Western blotting kit (Amersham Biosciences, Piscataway, NJ, USA) according to the manufacturer's protocol. Incubation of secondary antibody and anti-fluorescein was carried out both in presence of 1 × TBST with 2% nonfat dry milk. The fluorescent signals were scanned and documented using a Storm 860 scanner (Molecular Dynamics, Sunnyvale, CA, USA).

Antibodies against phospho-specific Stat3 (p-Stat3) (Tyr705 and Ser727), total Stat3, phospho-specific Stat1 (p-Stat1) (Tyr701), phospho-specific Stat5 (p-Stat5) (Tyr694) (Cell Signaling Technology, Beverly, MA, USA) were used to detect corresponding proteins on the membrane, respectively. A monoclonal antibody against glyceraldehyde-3-phosphate dehydrogenase (GAPDH; Chemicon International, Temecula, CA, USA) and a monoclonal antibody recognising FLAG (Sigma, St. Louis, MO, USA) were used to detect GAPDH as an internal protein loading control in all Western blots and to detect FLAG-tagged Stat3, respectively. To detect apoptosis, HeLa and SiHa cancer cells were transduced by either rAd/eGFP or rAd/dnStat3 for 2 days and then cell lysates were subjected to Western blotting using a monoclonal antibody that specifically recognises cleaved PARP (Asp214) (Cell Singling Tech. Danvers, MA, USA). The quantification of p-Stat3 (Tyr705) and p-Stat3 (Ser727) expressions was carried out using ImageQuan software (Molecular Dynamics, Sunnyvale, CA, USA). The expressions were normalised to GAPDH and shown as the percentages of p-Stat3 (Tyr705) in SiHa cells and p-Stat3 (Ser727) in RL95-2 cells.

### Transduction of dominant-negative Stat3 Y705F in cancer cells

The construction of recombinant adenovirus/CMVdnStat3 Y705F (rAd/dnStat3) is described previously (Kunisada *et al*, 1998). DnStat3 was generated from Stat3 by changing the Tyr at position 705 into phenylalanine. Its protein product cannot be activated through Tyr phosphorylation. The clone is tagged with a FLAG marker. About 2 × 10^5^ HeLa and SiHa cells were transduced with rAd/dNStat3 (Y705F) or rAd/eGFP (negative control viral vector) with multiplicities of infection (MOI) 400, 100, and 10. The cells were observed for cell growth and apoptosis at day 2 post-infection. Cell numbers of three random fields of views (magnification × 100) were scored for each rAd/dnStat3-transduced cells and negative controls in three independent experiments. For cell viability assay, about 1 × 10^4^ HeLa, SiHa, and Ishikawa cells were seeded in triplicates in 96-well plates with 100 *μ*l medium overnight and transduced with rAd/dnStat3 (Y705F) or rAd/eGFP (MOI=250). Cells were subjected to 3-(4,5-dimethylthiazol-2-yl)-2,5-diphenyl tetrazolium bromide (MTT) (Sigma-Aldrich, USA) cell viability assay 2 days later (Denizot and Lang, 1986). Cells were incubated with MTT (40 *μ*g ml^−1^) for 4 h and lysed in 20% SDS and 50% *N*,*N*-dimethylformamide (pH 4.5) and spectrophotometric reading was performed at OD_595_ by an EL808 Ultra Microplate Reader (Bio-Tek Instruments, Inc, Winooski, VT, USA).

### Cleaved caspase 3 immunofluorescent staining

About 1 × 10^5^ cells (HeLa and SiHa) were seeded on sterile coverslips in a six-well plate overnight. The cells were transduced by either rAd/eGFP or rAd/dnStat3 for 2 days and then fixed using methanol/acetone (v:v=1:1). The cells incubated with 5 and 10 *μ*M JSI-124 (cucurbitacin I) (EMD Biosciences, Inc. La Jolla, CA, USA), a JAK/Stat3 inhibitor, and DMSO (1:1000 dilution) served as positive and negative controls, respectively (Blaskovich *et al*, 2003). The cells were fixed using methanol/acetone after 24 h. Three washes followed the fixation using 1 × PBS buffer. During the third wash, the coverslips were transferred to a new six-well plate. For immunofluorescent staining, the cells were blocked in 1 × PBS with 10% normal horse serum for 1 h and incubated with primary rabbit antibodies that recognise cleaved-caspase-3 (Asp175) (Cell Singling Tech., Danvers, MA, USA) with 1:100, 1:50, and 1:100 dilution, respectively. Excess antibodies were removed using three washes of 1 × PBS with constant agitation, 10 min for each wash. Secondary goat anti-rabbit IgG(H+L) Alexa Fluor 594 antibody (Invitrogen, Carlsbad, CA, USA) (1:1000 dilution) was incubated with 1% bovine serum albumin in 1 × PBS for 1 h at RT. Unbound antibody was washed off three times using 1 × PBS. Nuclei were counterstained using 4′-6-diamidino-2-phenylindole (DAPI) (100 ng/ml) in distiled H_2_O for 5 min, and then rinsed three times with 1 × PBS, 10 min for each wash. The fluorescence and phase-contrast microscopic photographs were documented using LEICA DM-IRB inverted fluorescent microscope (Leica Microsystems, Inc., Bannockburn, IL, USA) with an attached Diagnostic RT-SE6 monochrome digital camera (Diagnostic Instruments, Inc, Sterling Heights, MI, USA).

## RESULTS

### Increased tyrosine phosphorylation of Stat3 in endometrial and cervical cancer tissues

**Figure 1:**
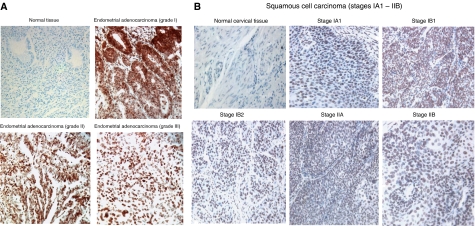
Expression of Stat3 phosphorylation (Tyr705) in (**A**) endometrial cancer tissues and (**B**) cervical cancer tissues. Endometrial and cervical cancer tissue microarray slides were stained using IHC methods and an IHC-validated phospho-specific Stat3 antibody (Tyr705). The representative examples of phosphorylated Stat3 staining in normal tissues and positive staining of endometrial cancer tissues (grades I, II and III) are shown. The representative examples of phosphorylated Stat3 staining in normal tissues and positive staining of cervical cancer tissues (different stages) are shown.

A total of 115 endometrial cancer specimens and five normal endometrial specimens were included on the microarray slides. Most of the endometrial cancer patients are between ages 46 and 80 and grades I and II (Supplementary Table 1). The most common cancer type in these tissue microarray slides was endometrial adenocarcinoma. We first examined the phosphorylation of Stat3 in endometrial cancer specimens. Our results showed that elevated levels of the phosphorylation (Tyr705) of Stat3 protein were detected in the nuclei of 24 out of 115 total endometrial cancer specimens (Supplementary Table 1). The representative examples of phosphorylated Stat3 staining in normal tissues and positive staining of endometrial cancer tissues (grades I, II, and III) are shown in Figure 1A. Positive p-Stat3 staining (Scores 2 and 3) were detected in 11.8% of patients in grade I, 25.8% of patients in grade II, and 27.3% of patients in grade III (Supplementary Table 1). Normal endometrial tissues express very low levels of phosphorylated Stat3 (Figure 1A). These results suggest that activation of Stat3 (Tyr 705) in endometrial cancer may be as early as at grade I but there is a slight increase in incidence at grades II and III.

**Figure 2:**
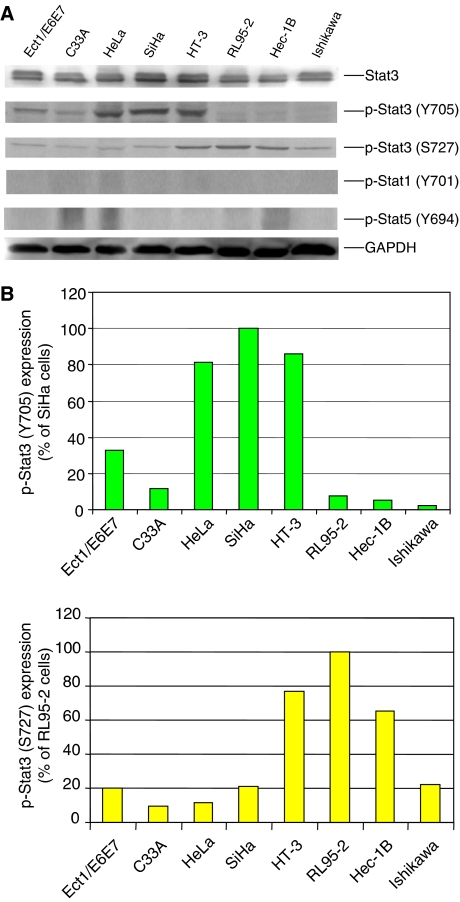
(**A**) Expression of Stat3, Stat1 and Stat5 phosphorylation in human immortalised cervical cell line (Ect1/E6E7) and cervical and endometrial cancer cell lines. A total of 100 *μ*g of total protein of cell lysates from various cell lines were resolved on 8% SDS–PAGE and subjected to Western blot analysis using antibodies that recognise phospho-specific Stat3 (Tyr705), Stat3 (Ser727), Stat1 (Tyr701), Stat5 (Tyr694), and GAPDH, respectively. (**B**) Densitometric quantitation of phospho-Stat3 (Tyr705) and Stat3 (Ser727) expressions. The phospho-Stat3 expressions are normalised to GAPDH expression in each cell line and shown in percentage of phospho-Stat3 (Tyr705) in SiHa cells and phospho-Stat3 (Ser727) in RL95-2 cells, respectively.

We next examined the phosphorylation of Stat3 (Tyr705) in cervical cancer specimens. A total of 104 cervical cancer specimens and seven normal cervical specimens were included in two independent microarray slides. Most of the cervical cancer patients are between ages 25 and 60 and the most common cervical cancer type in these two tissue microarray slides was squamous cell carcinoma. One microarray slide contains grade but no stage information and most of the cervical cancer patients are grades II and III (Supplementary Table 2). The other microarray slide contains stage but no grade information (Supplementary Table 2). Our results demonstrated that elevated levels of phosphorylation (Tyr705) of Stat3 protein were detected in the nuclei of 25 out of 104 total cervical cancer specimens (Supplementary Table 2). The representative examples of phosphorylated Stat3 staining in normal tissues and positive staining of cervical cancer tissues (different stages) are shown in Figure 1B. Positive phospho-Stat3 staining was detected in 10.8% of patients in grade II and 10.5% of patients in grade III (Supplementary Table 2). These results suggest that activation of Stat3 in cervical cancer can be detected at grades II and III. We only have one specimen in grade I that is negative, so the sample number is too small to draw conclusion for grade I. We also observed the elevation of Stat3 phosphorylation from Stage 0 to IIB and even detected two out of three squamous cell carcinoma *in situ* (TisN0M0) are positive (Supplementary Table 2). The elevation of Stat3 phosphorylation is also detected in cervical cancer with or without regional lymph node metastasis (Supplementary Table 2). These results suggest that the activation of Stat3 can be detected throughout all different stages and the activation of Stat3 may be a very early event in cervical cancer. Normal cervical tissues express very low levels of phosphorylated Stat3 (Figure 1B). We also examined the phosphorylation of Stat3 in human endometrial cancer cell lines, Ishikawa, RL-95-2, Hec-1B, and cervical cancer cell lines, C33A, HeLa, SiHa and HT-3. Human papillomavirus-immortalised cervical cell line (Ect1/E6E7), C33A and all three endometrial cancer cell lines, RL-95-2, Ishikawa, and Hec-1B express low or detectable levels of Stat3 phosphorylation. However, elevated phosphorylation of Stat3 (Tyr705) was detected in HeLa, SiHa, and HT-3 cervical cancer cell lines (Figure 2A and B). This is consistent with previous results that HT-3 cervical cancer cell line expressed elevated levels of Stat3 phosphorylation (Page *et al*, 2000).

Elevated Stat3 phosphorylation at Ser residue 727 (Ser727) was also detected in endometrial (18.3%, 11/60) and cervical (23.0%, 24/104) cancer tissues and cell lines (HT3, RL95-2 & Hec-1B) (Figure 2A and B; Tables 1 and 2). The frequencies of elevated p-Stat3 (Ser727) in both types of cancer tissues examined were very similar to the frequencies of p-Stat3 (Tyr705), 20.8% and 24.0%, respectively. Concurrence of elevated p-Stat3 (Tyr705) and p-Stat3 (Ser727) is high with 76.9% (20/26) and 63.6% (7/11) in cervical and endometrial cancers, respectively. HT-3 cell line was the only cell line to show high levels of expression of both p-Stat3 (Tyr705) and p-Stat3 (Ser727). HeLa and SiHa express high levels of p-Stat3 (Tyr705) but low levels of p-Stat3 (Ser727). In contrast, RL95-2 and Hec-1B express low levels of p-Stat3 (Tyr 705) but high levels of p-Stat3 (Ser727).

Stat1 and Stat5 are also important STAT members related to malignancies (Yu and Jove, 2004). The statuses of p-Stat1 (Tyr701) and p-Stat5 (Tyr694) in the cancer cell lines were also examined using Western blot analysis. None of eight cell lines showed signs of elevated phosphorylation for Stat1 but slightly elevated expression of p-Stat5 (Tyr694) was detected in C33A, HeLa and Hec-1B cells.

### Activated Stat3 is associated with increased expression of antiapoptotic genes in endometrial and cervical cancer tissues

Activation of Stat3 usually leads to the expression of downstream genes, *Bcl-xL*, *survivin*, and *Mcl-1*, that are important for cancer cell survival and chemoresistance. Immunohistochemistry was carried out to detect the expression of p-Stat3 (Ser727), *Bcl-xL*, *survivin*, and *Mcl-1* in both endometrial and cervical cancer tissues (Tables 1 and 2). Except for *Mcl-1* expression in cervical carcinomas, the expressions of these genes are associated with elevated p-Stat3 (Tyr705) with statistic significance (*P*<0.05). This implied that activation of Stat3 promotes the expression of antiapoptotic genes in these two types of cancers.

### rAd-mediated transduction of dnStat3 in cervical cell lines

**Figure 3:**
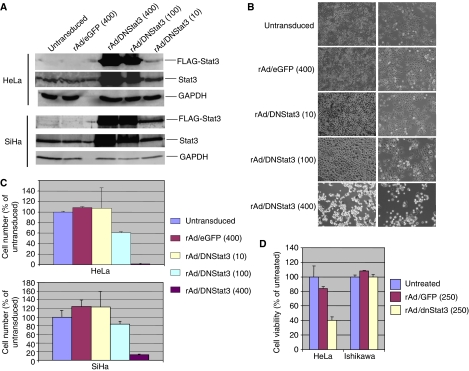
Transduction of dnStat3 inhibits cell growth and viability in cervical cancer cell lines. (**A**) Expression of dnStat3 mediated by rAd vector in a dose-dependent manner in HeLa and SiHa cervical cancer cell lines. One hundred micrograms of cell lysates were analysed on 10% PAGE analysis and then subjected to immunoblots probed with anti-FLAG, -Stat3 and – GAPDH antibodies. The dose-dependent expression of FLAG-tagged dnStat3 is according to the increased expression levels of total Stat3 protein. There was no detectable FLAG-tagged dnStat3 in cell lysates of untransduced or cells transduced with rAd/eGFP. (**B**) and (**C**) dnStat3 inhibits HeLa and SiHa cell growth. HeLa and SiHa cells were transduced with either rAd/dnStat3 or rAd/eGFP (MOI=10–400). Representative phase-contrast images of HeLa and SiHa cells 48 h post-transduction were shown at magnification × 100. Only representative data (MOI=400) are shown for cells transduced with rAd/eGFP for cells transduced with MOI lower than 400 showed no adverse effects on cell growth. Cells in five random individual microscopic fields (× 100) were scored on day 2 post-transduction of rAd/eGFP or rAd/dnStat3. The cell growth is shown in cell density/control (%). The averages and standard deviations are based on triplicate independent experiments. (**D**) dnStat3 reduces cell viability of cancer cells with elevated phospho-Stat3 at 48 h post-transduction. HeLa and Ishikawa cells were transduced with rAd/eGFP and rAd/dnStat3 (MOI=250). Cell viability was analysed using MTT assay.

Since Stat3 activation may play a role in promoting cell growth and survival in cervical and endometrial cancer cells, we subsequently investigated whether the interference of Stat3 activation by transduction of rAd/dnStat3 would affect cell growth or survival of cervical cancer cells *in vitro*. We introduced dnStat3 into HeLa and SiHa cervical cancer cell lines using an adenoviral vector delivery system. HeLa and SiHa cells were infected with rAd/dnStat3 (MOI=10, 100, and 400). FLAG-tagged dnStat3 expression levels in cervical cancer cells were detected in Western blots probed with an anti-FLAG antibody. Two days post-infection, dnStat3 was expressed in HeLa and SiHa cells in a dose-dependent manner, but not in untransduced cells and cells transduced with rAd/eGFP (MOI=400) (Figure 3A). The transduction efficiency of rAd vector on these cells was determined by infection of rAd/eGFP. More than 90% of cancer cells showed green fluorescence by day 2 post-infection with rAd/eGFP at a MOI of 400 (data not shown).

### Differential dnStat3-mediated suppression on cell growth of cervical cancer cells with elevated expression of p-Stat3 (Tyr705)

Cell growth of cervical cancer cells with elevated p-Stat3 (Tyr705) was differentially suppressed in the presence of dnStat3. Cervical cancer cells were transduced with either rAd/eGFP or rAd/dnStat3 (MOI=10–400) (Figure 3B). Growth of cells with/without transduction was normalised to untransduced controls at day 2 post-transduction (Figure 3C). The growth rates of untransduced cells were set at 100%. There were no adverse effects by rAd/eGFP on cell growth as observed in HeLa and SiHa cervical cancer cells when MOI of 400 were used. Cells transduced with rAd/eGFP (MOI=10 or 100) also showed no adverse effect in cell growth (data not shown). When transduced with rAd/dnStat3 (MOI=400), HeLa and SiHa cells showed dramatic decrease in cell growth with less than 5% and 20% of untransduced control HeLa (Figure 3A) and SiHa (Figure 3B) cells, respectively.

To investigate the selectivity of dnStat3-mediated cell growth inhibition, HeLa, SiHa and a non-p-Stat3 (Tyr705) expressing cell line, Ishikawa, were transduced with rAd/GFP and rAd/dnStat3 (MOI=250). Although all three endometrial cancer cell lines express non-detectable or very low levels of Stat3 phosphorylation (Tyr705), we tested Ishikawa rather than RL95-2 and Hec-1B cell line because Ishikawa cell line expresses lowest levels of Stat3 phosphorylation at Ser residue 727. MTT assay indicated that cell viability of Ishikawa cells is not affected by dnStat3 expression whereas HeLa (Figure 3D) and SiHa cell (data not shown) viability was decreased.

### Apoptosis induced by dnStat3 and a JAK/Stat3 small molecular inhibitor through caspase-3 pathway

**Figure 4:**
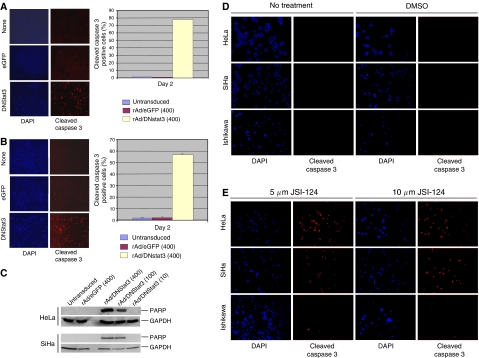
Transduction of dnStat3 induces apoptosis through caspase 3 pathway in (**A**) HeLa and (**B**) SiHa cervical cancer cell lines day 2 post-transduction. Cells were transduced with rAd/dnStat3 or rAd/eGFP (MOI=400). Cells incubated with JSI-124 (5 and 10 *μ*M), a Stat3 inhibitor, and DMSO served as a positive and negative control, respectively. Cells were fixed in methanol/acetone (v : v=1 : 1) and then immunostained with anti-cleaved caspase-3 antibody after 48 h. Cleaved caspase-3 immunoreactivies were observed in cells transduced with rAd/dnStat3, but much less in cells transduced with rAd/eGFP or the untransduced controls. Magnifications of all images were × 100. The percentages of cleaved caspase-3 positive cells were scored in five microscopic fields. The averages and standard deviations are based on three independent experiments. (**C**) dnStat3 induces cleavage of PARP in HeLa and SiHa cell lines. HeLa and SiHa cells were transduced with rAd/dnStat3 and rAd/eGFP (MOI:10, 100 and 400). After 48 h, cell lysates (100 *μ*g) were fractionated using SDS–PAGE and subject to Western blots probed with anti-cleaved PARP and -GAPDH antibodies. Cleaved caspase 3: anticleaved-caspase-3 antibody immunofluorescent staining; DAPI: nuclear staining with DAPI; none: untransduced cells.

Loss of cell numbers in cells transduced with rAd/dnStat3 suggested cell death as the cause. We explored whether apoptosis may contribute to the cell death. To investigate the mechanism underlying the cervical cancer cell death, HeLa and SiHa cells were fixed at day 2 post-transduction of rAd/eGFP or rAd/dnStat3 and then subjected to immunofluorescent staining using an antibody that recognises cleaved caspase 3 or Western blot analysis using antibody that recognises cleaved PARP for apoptosis detection. As expected, rAd/dnStat3 inhibited Stat3 pathway and induced cleavage of caspase 3 in both cell lines (Figure 4A and B). There were basal levels of cleaved caspase-3 in negative controls (untransduced, transduced with rAd/eGFP, or DMSO sham) in HeLa and SiHa cancer cells lines. However, apoptosis induced by the expression of dnStat3 was evidenced as caspase 3 and PARP were observed in dnStat3-expressing HeLa and SiHa cervical cancer cell lines (Figure 4A–C). Furthermore, a small molecule inhibitor that targets JAK/Stat3, JSI-124, also more selectively induced cleavage of caspase 3 in HeLa and SiHa cancer cell lines than Ishikawa cell line, which has low expression of elevated levels of Stat3 phosphorylation (Tyr705) (Figure 4E).

## DISCUSSION

Endometrial cancer is the most common gynecologic malignancy in developed countries, with approximately 40 000 new diagnoses each year in the United States alone. There are about 10 370 new cases of invasive cervical cancer in the United States and about 3710 women will die from this disease. Constitutive Stat3 signaling appears to play a role in the cell transformation and tumour progression by stimulating cell growth, promoting tumour angiogenesis, mediating immune evasion and conferring resistance to apoptosis induced by chemotherapeutic agents (Niu *et al*, 2002; Real *et al*, 2002; Wei *et al*, 2003; Wang *et al*, 2004). However, whether Stat3 is activated in endometrial and cervical cancers is not known. The present study investigated the activation Stat3 in endometrial and cervical carcinomas, which should help us to better understand the cancer progression of endometrial and cervical cancers that involve the activation of multiple oncogenic pathways including the constitutive Stat3 pathway. The elevated levels of Stat3 phosphorylation were detected in 25.2% of 107 total cervical cancer specimens (Supplementary Table 2) as well as three out of four human cervical cancer cell lines. Although none of the three endometrial cancer cell lines we studied expressed elevated Stat3 phosphorylation, 20.8% of 115 total endometrial cancer specimens showed elevated levels of Stat3 phosphorylation (Supplementary Table 1). Our previous results also indicated that Stat3 is activated in ovarian cancer (Huang *et al*, 2000). Therefore, constitutive activation of Stat3 seems to be one of the common molecular mechanisms to promote oncogenesis in gynecologic cancers including ovarian, endometrial, and cervical cancers. We observed that Stat3 is activated in cervical cancer early stages such as Stage 1, suggesting that Stat3 is a target of chemoprevention in cervical cancer. Inhibition of activated Stat3 in early stage(s) of cervical cancer might prevent further progression of cervical cancer. How Stat3 is activated in endometrial cancer is currently unknown and it will be of interest to further examine the mechanisms. Stat3 activation in cervical cancer is also largely unclear but one of the mechanisms may be involved in interleukin-6 (Wei *et al*, 2003). Immunohistochemistry on tissue arrays and Western blots of cell lines showed that Stat3 activation through phosphorylation at Ser 727 might be important for endometrial and cervical malignancies. Stat1 and Stat5 might not be crucial for these cancers since phosphorylation of these two STAT members are not evident.

Since Stat3 phosphorylation at Tyr705 is elevated in cervical and endometrial cancer tissues and Stat3 pathway has been shown to participate in oncogenesis (Bromberg *et al*, 1999; Bowman *et al*, 2000), we examined whether the inhibition of Stat3 pathway by dnStat3 in cervical cancer cells could suppress cancer cell growth. Suppression of cell growth was observed in cervical cancer cells expressing dnStat3, whereas growth of untransduced cells or transduced with rAd/GFP remained unaffected. This indicates that the growth inhibition of cervical cancer cells is a dnStat3-dependent inhibitory effect. Transduction of dnStat3 also induces apoptosis in cervical cancer cells *in vitro*. Apoptosis caused by dnStat3 is apparently through the caspase 3-dependent pathway, since cleaved caspase 3 was detected in cancer cells expressing dnStat3 or treated with JSI-124 as demonstrated by immunofluorescent staining. Increased expressions of antiapoptotic *Bcl-xL*, *survivin* and *Mcl-1* are strongly associated with the elevated p-Stat3 (Tyr705) in cervical and endometrial cancer tissues. They are the candidate targets by dnStat3 for its cell growth inhibition and apoptosis induction. Our data strongly support the possibility that the activated Stat3 pathway could serve as a therapeutic target in cervical and possibly in endometrial cancers using a dominant-negative Stat3 mutant or small molecular inhibitors.

Other methods that targets the Stat3 signaling pathway in cancer cells have also been explored, which include using anti-sense RNA (Grandis *et al*, 2000; Epling-Burnette *et al*, 2001; Calvin *et al*, 2003; Chiarle *et al*, 2005), siRNA (Konnikova *et al*, 2003; Lee *et al*, 2004), small molecules (Song *et al*, 2005; Turkson *et al*, 2005) and decoy-oligos (Leong *et al*, 2003; Chan *et al*, 2004).

In summary, our results demonstrated for the first time that Stat3 phosphorylation is elevated in clinical human endometrial and cervical cancer samples. Stat3 appears to be one of the oncogenic pathways activated in human endometrial and cervical cancers. The constitutive Stat3 signaling may be a novel therapeutic target for cancer intervention in cervical and endometrial carcinomas.

## Figures and Tables

**Table 1:** The association of p-Stat3 (Tyr705) with the expression of p-Stat3 (Ser727) and potential Stat3 downstream targets in endometrial carcinomas

		**IHC – staining positive**		**Association with P-Stat3 (Tyr705)^a^**		
	**Total number of cancer tissues**	**Numbers**	**(%)**	**Numbers^a^**	**(%)**	**Pearson χ^2^ test^b^**
P-Stat3 (Tyr705)	60	14	(23.3)	14	(100)	
P-Stat3 (Ser727)	60	11	(18.3)	7	(63.6)	*P*<0.05
*Bcl-xL*	60	21	(35.0)	13	(61.9)	*P*<0.05
*Survivin*	60	14	(23.3)	10	(71.4)	*P*<0.05
*Mcl-1*	60	21	(35.0)	12	(57.1)	*P*>0.05

a The numbers associated with P-Stat3 (Tyr705) divided by total numbers of IHC-staining positive.

b *P*<0.05 is considered as statistically significant.

**Table 2:** The association of p-Stat3 (Tyr705) with the expression of p-Stat3 (Ser727) and potential Stat3 downstream targets in cervical carcinomas

		**IHC – staining positive**		**Association with P-Stat3 (Tyr705)^a^**		
	**Total numbers of cancer tissues**	**Numbers**	**(%)**	**Numbers^a^**	**(%)**	**Pearson χ^2^ test^b^**
P-Stat3 (Tyr705)	104	25	(24.0)	25	(100)	
P-Stat3 (Ser727)	104	24	(23.0)	18	(75.0)	*P*<0.05
*Bcl-xL*	104	27	(25.9)	20	(74.0)	*P*<0.05
*Survivin*	55	10	(18.2)	6	(60.0)	*P*<0.05
*Mcl-1*	55	16	(29.0)	10	(62.5)	*P*<0.05

a The numbers associated with p-Stat3 (Tyr705) divided by total numbers of IHC-staining positive.

b *P*<0.05 is considered as statistically significant.
