# Supplementary material for: Stat3 activation in human endometrial and cervical cancers
Source: Br J Cancer. 2007 Feb 20;96(4):591–9. doi: 10.1038/sj.bjc.6603597 (PMC2360038; doi:10.1038/sj.bjc.6603597)
Supplement: Supplementary Tables [file 6603597x1.doc]

| **Supplemental Table 1. Clinicopathological parameters of endometrial carcinomas analyzed.** | | | | |
| --- | --- | --- | --- | --- |
| Clinicopathological parameters | |  | Numbers (%)^a^ | # of P-Stat3 positive (%)^b^ |
| Gender | Female | | 115 (100) | 24 (20.8) |
|  |  | |  |  |
| Age (years) | 31-45 | | 9 (7.8) | 1 (11.1) |
|  | 46-60 | | 72 (62.6) | 14 (19.4) |
|  | 61-80 | | 34 (29.6) | 9 (26.5) |
|  | Mean | | 57 | 8 (14.0) |
|  | Median | | 55 | 9 (16.4) |
| Grade (Total 115) | I | | 34 (29.6) | 4 (11.8) |
|  | II | | 66 (57.4) | 17 (25.8) |
|  | III | | 11 (9.6) | 3 (27.3) |
|  | No Grade | | 4 (3.5) | 0 (0) |
|  |  | |  |  |
| Histology (Total 115) | Endometrial adenocarcinoma | | 111 (96.5) | 23 (20.7) |
|  | Adenosquamous carcinoma | | 3 (2.6) | 0 (0) |
|  | Endometrial papillary adenocarcinoma | | 1 (0.9) | 1 (100) |
|  |  | |  |  |
|  |  | |  |  |

^a^ The percentages were determined by individual number divided by total numbers (115).

^b^  The percentages were determined by the numbers of positive P-Stat3 staining (Tyr705) divided by the corresponding numbers on the left columns.

| **Supplemental Table 2. Clinicopathological parameters of cervical carcinomas analyzed.** | | | |
| --- | --- | --- | --- |
| Clinicopathological parameters |  | Numbers (%) | P-Stat3 positive (%)^l^ |
| Gender | Female | 104 (100) | 25 (24.0) |
|  |  |  |  |
| Age (Years, total 104) | 25-40 | 24 (23.0) ^i^ | 5 (20.8) |
|  | 41-60 | 63 (60.5) | 17 (27.0) |
|  | 61-70 | 15 (14.4) | 2 (13.3) |
|  | Mean | 49 | 9 (18.4) |
|  | Median | 48 | 7 (14.6) |
|  |  |  |  |
| Grade (Total 57) | I | 1 (1.8) ^j^ | 0 (0) |
|  | II | 37 (64.9) | 4 (10.8) |
|  | III | 19 (33.3) | 2 (10.5) |
|  |  |  |  |
| Stage (Total 47) |  |  |  |
|  | I ^a^ | 3 (6.3) | 2 (66.7) |
|  | IA1^b^ | 4 (8.5) | 2 (50.0) |
|  | IB^a^ | 2 (4.2) | 1 (50.0) |
|  | IB1^c^ | 22 (46.8) | 8 (36.4) |
|  | IB2^d^ | 11 (23.4) | 4 (36.4) |
|  | IIA^e^ | 3 (6.3) | 1 (33.3) |
|  | IIB^f^ | 2 (4.2) | 1 (50.0) |
|  |  |  |  |
| Metastasis in regional lymph nodes | N0^g^ | 28 (59.5) ^k^ | 13 (46.4) |
| (Total 50) | N1^h^ | 19 (40.4) | 8 (42.1) |
|  |  |  |  |
| Histology (Total 104) | Squamous cell carcinoma | 92 (88.4)^i^ | 27 (29.3) |
|  | Microinvasive squamous cell carcinoma | 5 (4.8) | 4 (80.0) |
|  | Adenosquamous carcinoma | 5 (4.8) | 1 (20.0) |
|  | Adenocarcinoma | 2 (1.9) | 0 (0) |
|  |  |  |  |
|  |  |  |  |

^a^ I, IB – Information for more specific staging not available

^b^ IA1 – The area of invasion is < 3mm deep and <7mm wide

^c^ IB1 – The cancer is ≤ 4cm in size

^d^ IB2 – The cancer is > 4 cm in size

^e^ IIA – The cancer has spread to the upper part of the vagina

^f^ IIB – The cancer has spread to the parametrial tissue

^g^ N0 - No regional lymph node metastasis

^h^ N1 - Metastasis in 1 to 3 regional lymph nodes

^i^ The percentages were determined by individual number divided by total numbers (104).

^j^ This microarray slide contains 57 cancer specimens and only have grades but no stages information. The percentages were determined by individual number divided by total numbers.

^k^ This microarray slide contains 50 cancer specimens and only have stages but no grades information. The percentages were determined by individual number divided by total numbers.

^l^  The percentages were determined by the numbers of positive P-Stat3 staining (Tyr705) divided by the corresponding numbers on the left columns.
